# Economic Perspective of the Use of Wearables in Health Care: A Systematic Review

**DOI:** 10.1016/j.mcpdig.2024.05.003

**Published:** 2024-05-14

**Authors:** Gioacchino D. De Sario Velasquez, Sahar Borna, Michael J. Maniaci, Jordan D. Coffey, Clifton R. Haider, Bart M. Demaerschalk, Antonio Jorge Forte

**Affiliations:** a Division of Plastic Surgery, Mayo Clinic, Jacksonville, FL; b Division of Hospital Internal Medicine, Mayo Clinic, Jacksonville, FL; c Center for Digital Health, Mayo Clinic, Rochester, MN; d Department of Physiology and Biomedical Engineering, Mayo Clinic, Rochester, MN; e Department of Neurology, Mayo Clinic College of Medicine and Science, Phoenix, Arizona; f Institute for Reconstructive Surgery, Houston Methodist Hospital, Houston, TX

## Abstract

The objective of this study is to explore the current state of research concerning the cost-effectiveness of wearable health technologies, excluding hearing aids, owing to extensive previous investigation. A systematic review was performed using PubMed, EMBASE/MEDLINE, Google Scholar, and Cumulated Index to Nursing and Allied Health Literature to search studies evaluating the cost-effectiveness of wearable health devices in terms of quality-adjusted life years and incremental cost-effectiveness ratio. The search was conducted on March 28, 2023, and the date of publication did not limit the search. The search yielded 10 studies eligible for inclusion. These studies, published between 2012 and 2023, spanned various locations globally. The studies used data from hypothetical cohorts, existing research, randomized controlled trials, and meta-analyses. They covered a diverse range of wearable technologies applied in different health care settings, including respiratory rate monitors, pedometers, fall-prediction devices, hospital-acquired pressure injury prevention monitors, seizure detection devices, heart rate monitors, insulin therapy sensors, and wearable cardioverter defibrillators. The time horizons in the cost-effectiveness analyses ranged from less than a year to a lifetime. The studies indicate that wearable technologies can increase quality-adjusted life years and be cost-effective and potentially cost-saving. However, the cost-effectiveness depends on various factors, such as the type of device, the health condition being addressed, the specific perspective of the health economic analysis, local cost and payment structure, and willingness-to-pay thresholds. The use of wearables in health care promises improving outcomes and resource allocation. However, more research is needed to fully understand the long-term benefits and to strengthen the evidence base for health care providers, policymakers, and patients.

**Article Highlights**

• This article provides a systematic review of economic evaluations of wearable health technologies in health care, highlighting their variability in cost-effectiveness and impact across different global settings and medical conditions.
• The findings illustrate that wearable technologies can significantly improve health care outcomes, particularly in terms of increasing quality-adjusted life years, across various patient demographic characteristics and conditions.
• The study discusses the multiple factors influencing the cost-effectiveness of wearables, including the type of technology, targeted health conditions, economic perspectives, and local cost structures.
• The authors emphasize the need for further comprehensive research to solidify the evidence on the economic benefits of wearables, suggesting future studies explore longer time horizons and include a broader range of wearable technologies.
• The review advocates for the integration of wearable technologies into health policy and practice, underscoring the potential for optimized resource allocation and improved patient outcomes with adequate data privacy protection.

The advent and proliferation of wearable technology in health care have opened a plethora of opportunities for continuous health monitoring, early disease detection, and personalized care delivery.^1^ These devices, often unobtrusive and user-friendly, have made it possible to monitor a range of health parameters, including vital signs, physical activity, and sleep patterns in real-time.^2^

The health care wearables market is vast and diverse, including fitness trackers, smartwatches, biosensors, and smart clothing.^3^, ^4^, ^5^, ^6^ Although the potential benefits of these devices in improving health outcomes could be substantial, they also represent an important economic impact in terms of cost and resource allocation.^7^ Consequently, a comprehensive evaluation of the cost-benefit of using wearable devices in health care is crucial to inform decision-making for health care providers, policymakers, insurers, and patients.^8^

Quality-adjusted life years (QALYs) is a recognized measure that quantifies the benefit of a health intervention by considering both the quantity and quality of life.^8^^,^^9^ Despite its extensive use in health economics, there is a dearth of comprehensive reviews exploring the cost-benefit of wearables in health care through a QALY lens.^10^^,^^11^ Moreover, the current literature is dominated by economic impact studies of hearing aids, necessitating a more diverse focus.^12^, ^13^, ^14^, ^15^

This systematic review aims to address this gap in the literature by reviewing studies on the cost-benefit analysis of wearable health technologies, excluding hearing aids, and focusing specifically on wearables that monitor vital signs, hemodynamics, and other nonimplantable devices. The objective is to provide a comprehensive review of the existing literature on the economic impact of the advent of wearables as valuable tools for health care professionals, which could also serve as a valuable resource for stakeholders interested in the cost-effectiveness and health benefits of implementing wearable technologies in health care.

## Patients and Methods

### Eligibility Criteria

#### Population and Interventions

Our study included journal articles evaluating the cost-effectiveness of wearables devices undertaken with any time horizon. Studies were eligible for inclusion if they assessed the cost-effectiveness as an outcome in terms of QALYs. An additional inclusion criterion was the report of incremental cost-effectiveness ratio (ICER) when compared with a standard of care, other interventions, or no intervention. We did not limit our study’s inclusion criteria per object population age, country, or currency to the costs reported. We excluded studies with insufficient data for analysis, cost-analysis only on hearing aids, review articles, and conference papers.

#### Information Sources and Search Strategy

**Figure 1:**
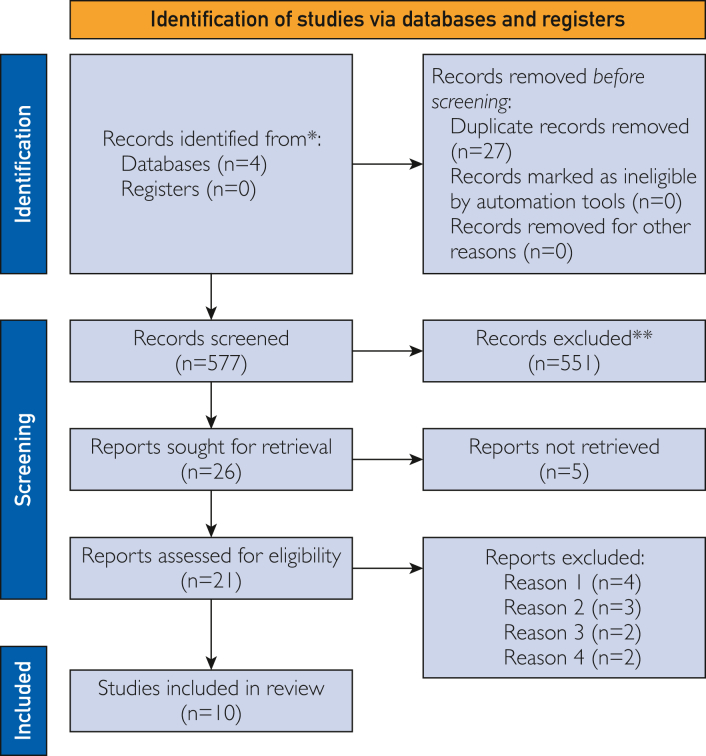
Study selection process: process of study selection after the PRISMA 2020 flow diagram for systematic reviews. PRISMA, Preferred Reporting Items for Systematic reviews and Meta-Analyses.

We used 4 electronic databases to run our search: PubMed, EMBASE/MEDLINE, Google Scholar, and Cumulated Index to Nursing and Allied Health Literature, using Boolean expressions to create a complex search strategy. The search was performed on March 18, 2023, and the date of publication did not limit the search. The details of the search strategy can be found in Appendix 1 (available online at https://www.mcpdigitalhealth.org/). It must be highlighted that only the first 10 pages from the Google Scholar search were screened. We used the Preferred Reporting Items for Systematic reviews and Meta-Analyses 2020 statement as the basis of our organization (Figure 1).^16^

### Selection and Data Collection Process

The first 2 authors (G.D.S.V., S.B.) independently conducted the initial literature search. Any duplicate findings were detected and eliminated using the EndNote tool from Clarivate Analytics. The full text of the pertinent articles was obtained after a preliminary screening of the articles based on their titles and abstracts. These articles underwent a detailed review, adhering to the pre-established selection criteria. The decision of the third or fourth authors resolved any disputes between the initial 2 authors.

For each study, the following information was extracted and recorded: authors, year of publication, country, study goals, population, setting, eligibility criteria, duration of studies, interventions, demographic characteristics, type of wearable devices, control groups, total costs per intervention arm, QALYs, ICERs, cost-effectiveness thresholds or willingness-to-pay, probability of being cost-effective, limitation of the studies, funding, and conflicts of interests.

#### Risk of Bias of Individual Studies

**Figure 2:**
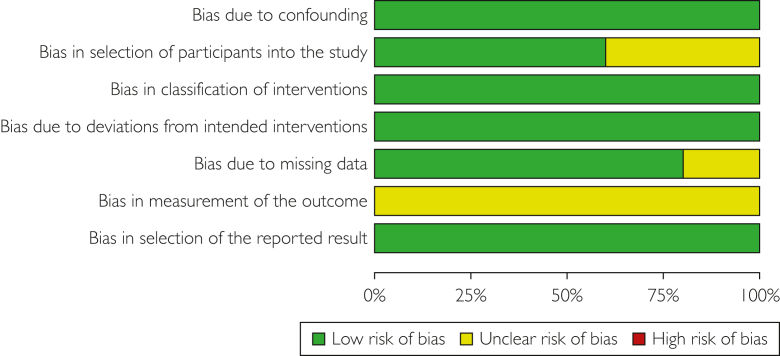
Risk of bias graph: review authors’ judgments about each risk of bias item presented as percentages across all included studies.

**Figure 3:**
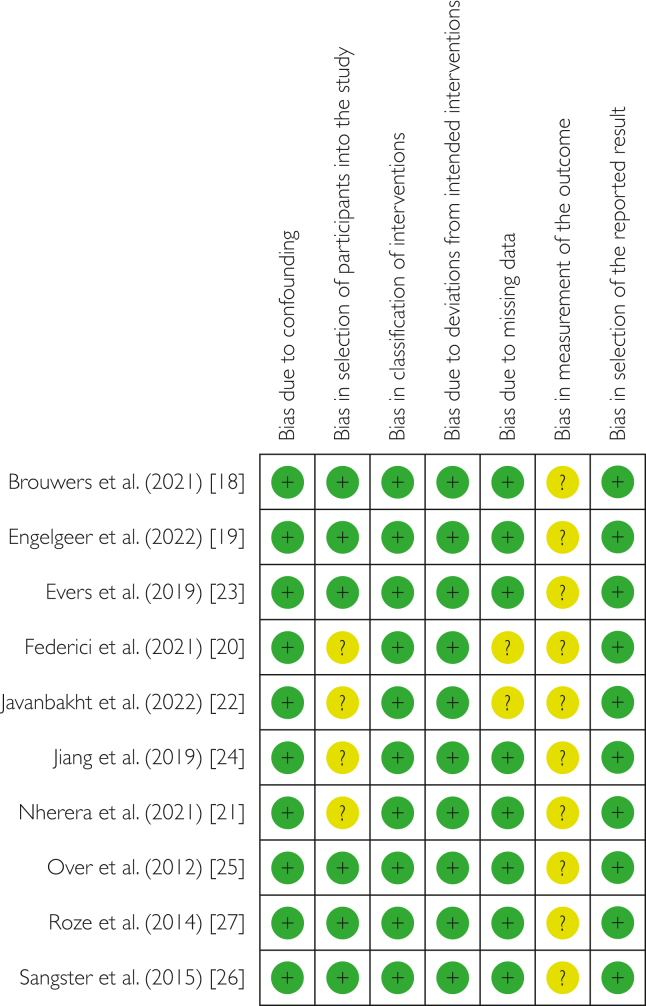
Risk of bias summary: review authors’ judgments about each included article’s risk of bias in the different components of the analysis. Red stands for high risk of bias, yellow stands for unclear risk, and green stands for low risk of bias.

Each study was independently evaluated for bias by the first 2 authors (G.D.S.V., S.B.) using the Risk Of Bias In Non-randomised Studies-I tool from the Cochrane Library for nonrandomized studies.^17^ The decision of the third or fourth authors addressed conflicts between the first 2 authors. Subsequently, a summary and a graph were created using RevMan 5.4 (Cochrane Collaboration), enabling the stratification of bias in diverse areas (Figures 2 and 3).

#### Synthesis of Results

After a comprehensive review of the included studies, the most significant data from the methodologies, used devices, outcomes, costs, and effectiveness were tabulated using the Microsoft Excel spreadsheet software to display the results of each study (Tables 1 and 2). Finally, the narrative synthesis was performed after tabulating the results, describing the differences between studies regarding their methodologies, interventions, and aims.

**Table 1:** Summary of Included Studies

Reference, year	Country	Type of wearable	Setting
Javanbakht et al,^22^ 2022	United Kingdom	RespiraSense (Respiratory rate monitor)	Patients with pneumonia
Over et al.^25^ 2012	Netherlands	Pedometer	Population with insufficient physical activity
Federici and Pecchia,^20^ 2021	United Kingdom	Fall-prediction device	Elderly patients with orthostatic hypotension
Nherera et al,^21^ 2021	United States	LEAF Patient Monitoring System (device for prevention of HAPIs)	Acutely ill patients
Engelgeer et al,^19^ 2022	Netherlands	NightWatch (Seizure detection device)	Pediatric patients with refractory epilepsy living at home
Brouwers et al,^18^ 2021	Netherlands	Wrist-worn heart rate monitor and a hip-worn triaxial accelerometer for cardiac telerehabilitation	Outpatient CR for the treatment of CAD
Sangster et al,^26^ 2015	Australia	Pedometer-based Coaching Programs: healthy weight and physical activity	Cardiac disease
Roze et al,^27^ 2015	Sweden	Sensor-augmented pump insulin therapy	Type 1 diabetes
Jiang et al,^24^ 2019	China	WCD	Interval between the removal and reinsertion of the ICD
Evers et al,^23^ 2020	United States	WCD	Pediatric patients with dilated cardiomyopathy before ICD placement

CAD, coronary artery disease; CR, cardiac rehabilitation; HAPI, hospital-acquired pressure injury; ICD, implantable cardioverter-defibrillator; WCD, wearable cardioverter-defibrillator.

**Table 2:** Cost-Effectiveness Analysis and Key Findings

Reference, year	Type of wearable	Control group	QALYs	Total costs	ICER	Control groups’ cost and QALYs	Willingness-to-pay	Probability of being cost-effective	Key findings
Javanbakht et al,^22^ 2022	RespiraSense (respiratory rate monitor)	IM	ARRM: 6.926 per patient	ARRM: £4752.0 per patient Incremental difference: £−221.4	ARRM was dominant	QALY: IM: 6.917 Costs IM: £4973 per patient	£20,000 per QALY	100%	The ARRM strategy using RespiraSense was less costly and more effective than IM. Results imply that combining IM with ARRM is likely a cost-saving intervention, as opposed to IM alone
Over et al,^25^ 2012	Pedometer	No intervention	5500 QALY gained	Total costs: €61.2 million	€11,100 per QALY gained	Not reported	€20,000 per QALY	66%	The study suggested that the pedometer scenario could lead to significant gains in life years and QALYs. However, the cost-effectiveness varied depending on the long-term effect of increased walking and comparisons with other interventions
Federici and Pecchia,^20^ 2021	Fall-prediction device	No intervention	0.035 QALYs per patient	Incremental costs of £518	£14,719	Not reported	£20,000 per QALY	Not reported	The study highlighted the importance of considering cost-effectiveness in the early stages of wearable device development for health care
Nherera et al,^21^ 2021	Patient Monitoring System for prevention of HAPIs	The international guidelines on pressure injury prevention (NPUAP/EPUAP 2019)	15.49 QALY per 1000 patients	$39,579,924 per 1000 patients	Not explicitly stated. The intervention arm was dominant (less costly [−$6,621,113] and more effective [+0.95 QALYs])	Costs = $46,201,037; QALYs = 14.54	$50,000 per QALY	99%	Wearable sensors resulted in improved outcomes (77% decrease in HAPIs) and an anticipated cost savings of $6621 per patient over a year
Engelgeer et al,^19^ 2022	NightWatch (seizure detection device)	No seizure detection device used	Mean 0.90 QALY per participant	€2463.03 per participant	€846 per patient	QALYs = 0.9; Costs = €3238	€50,000 per QALY	72%	The study determined that NightWatch led to cost savings and was cost-effective compared with no-use seizure detection devices. Additionally, it had similar effectiveness in improving caregivers’ quality of life
Brouwers et al,^18^ 2021	Wrist-worn heart rate monitor and a hip-worn triaxial accelerometer for CTR	CB-CR	Mean 0.841; *P*>.05 compared with CB-CR	€20,495 per participant per year; *P*>.05 compared with CB-CR	Comparable (CTR probably cost-effective compared with. CB-CR, but the difference was not significant)	QALY mean = 0.844; costs = €24,381 per participant	€20,000 per QALY	84%	CTR combined with relapse prevention is probably cost-effective compared with CB-CR, with comparable quality of life and nonsignificantly lower total costs
Sangster et al,^26^ 2015	Pedometer-based coaching programs: healthy weight and physical activity	Not compared with the standard of care	Healthy weight: 0.034 QALYs per participant Physical activity: 0.018 QALYs per participant	Healthy weight: $1260 per participant Physical activity: $2112 per participant	Healthy weight intervention dominant over physical activity intervention (less costly, more effective in QALY)	Not Applicable	$60,000 per QALY	96% for healthy weight over physical activity	Healthy weight intervention was more cost-effective, resulting in higher QALY gains (0.016 difference) and additional adjusted QALYs (0.007) after adjusting for baseline differences. Healthy weight was more effective and cost-saving for rural participants and noncardiac rehabilitation attendees
Roze et al,^27^ 2015	Sensor-augmented pump insulin therapy	Self-monitoring of plasma glucose + continuous subcutaneous insulin infusion	13.05 QALYs per participant (*P*>.05 compared with control)	2,872,525 SEK	367,571 SEK per QALY	QALYs: 12.29 Total costs: 2,592,563 SEK	500,000 SEK per QALY	>70%	Sensor-augmented pump use was associated with a decreased incidence of diabetes complications and an extended life expectancy
Jiang et al,^24^ 2019	WCD	1. Home group with no WCD 2. Inpatient group	3.0990 QALYs	$36.569 at a daily WCD cost of 24$	At a daily WCD cost of $24: 1) compared with home-no WCD: $22,540 2) compared with the inpatient: dominant	1) Home-no WCD: 3.0132 QALYs; costs: $34,635 2) Inpatient group: 3.0553 QALYs; costs: $36,780	$57,315 per QALY	Over 94% at a daily cost of <$48	The WCD was likely cost-effective when its daily cost was between $24 and $72. At the daily cost of $96, it exceeded the cost-effectiveness threshold, making hospital stay the preferred option
Evers et al,^23^ 2020	WCD	1. Home group with no WCD 2. Inpatient group	42.58	$569,757	$20,103 per QALY compared with the home group with no WCD	1. Home-no WCD: QALYs: 40.63; costs: $530,599 2. Inpatient QALYs: 42.99; costs: $676,957	$50,000 per QALY	Not reported	Despite predicting higher QALYs, the inpatient strategy was not cost-effective compared with home-no WCD, with an ICER of $255,652

ARRM, automated respiratory rate monitoring; CB-CR, center-based cardiac rehabilitation; CTR, cardiac telerehabilitation; EPUAP, European Pressure Ulcer Advisory Panel; HAPI, hospital-acquired pressure injury; ICER, incremental cost-effectiveness ratio; IM, intermittent nurse-led respiratory rate monitoring; NPUAP, National Pressure Ulcer Advisory Panel; QALY, quality-adjusted life year; SEK, Swedish krona; WCD, wearable cardioverter-defibrillator.

## Results

### Study Selection and Characteristics

The search provided 604 studies, yielding 577 after the removal of duplicates. After that, 551 were excluded after initial screening by titles and abstracts. Twenty-one full text articles of the 26 articles were retrieved and assessed, making 10 eligible for inclusion (Figure 1). From the excluded studies, 4 did not analyze QALYs on the use of wearables; 3 did not assess the total costs for each group; 2 were review articles, and 2 were found irrelevant.

The 10 included studies were published between 2012 and 2023, with 5 published within the past 3 years.^18^, ^19^, ^20^, ^21^, ^22^ The geographical distribution of the studies spanned the United Kingdom, Netherlands, the United States, Australia, Sweden, and China.

Four studies simulated cost-effectiveness analyses on hypothetical cohorts,^22^, ^23^, ^24^, ^25^ five drew on data from existing research or randomized controlled trials,^18^, ^19^, ^20^, ^21^^,^^26^ and one used data from a published meta-analysis.^27^

All studies were conducted on diverse population groups. Nine studied patients with specific medical conditions; notably, 4 of these specifically focused on individuals with cardiac conditions.^18^^,^^23^^,^^24^^,^^26^ Furthermore, 1 study focused on the general population attending general practitioner consultations.^25^ Two studies analyzed the cost-effectiveness of their respective wearable in pediatric patients^19^^,^^23^ while one study focused on the elderly population.^20^

Concerning the time horizons used in cost-effectiveness analyses, 5 studies opted for less than a year,^18^^,^^19^^,^^21^^,^^23^^,^^26^ and 4 studies with 5 years or more,^20^^,^^22^^,^^24^^,^^27^ with 2 among them extending to a lifetime.^22^^,^^27^ Notably, 1 study^25^ did not provide explicit information about its time horizon.

There was heterogeneity between the studies in methodology, population, time of data collected, tools and methodology for cost analyses, and end points. The types of wearables studied and the setting, even among those with the same type of wearable varied among studies. In general, the studies differed in the way they assessed the total costs and QALYs.

### Risk of Bias in Studies

The overall risk of bias was considered low to moderate, both within and across studies. Figures 2 and 3 show specific findings in each domain.

### Results for Individual Studies

The specific results for each study are detailed and organized in Tables 1 and 2. Javanbakht et al^22^ compared the cost-effectiveness of automatic respiratory rate monitoring with that of RespiraSense to intermittent nurse-led monitoring in pneumonia patients. The analysis used a decision tree followed by a Markov model to assess a hypothetical patient cohort and measure costs, length of stay, QALYs, and mortality. The study calculated the ICER and compared it with the UK cost-effectiveness threshold (£20,000 per QALY) to assess the economic value of automatic respiratory rate monitoring with RespiraSense in pneumonia management.

Over et al^25^ compared the use of a pedometer in insufficiently active patients with no intervention. Participants in each stage of the pedometer intervention were outlined. The “Rijksinstituut voor Volksgezondheid en Milieu (RIVM)” Chronic Disease Model was used to calculate the effects of the pedometer scenario on QALYs and health care costs. The study conducted a probabilistic sensitivity analysis to address parameter uncertainty.

Federici and Pecchia^20^ used the “Monitoring and Assessment Framework for the European Innovation Partnership on Active and Healthy Ageing (MAFEIP)” tool for early health technology assessment in a case study involving a wearable device for fall-prediction in elderly patients. They used the MAFEIP tool to estimate the device’s cost-effectiveness and conducted sensitivity analyses to assess its robustness.

Nherera et al^21^ used a Markov model to assess the cost-effectiveness of a wearable patient sensor along with standard care for preventing hospital-acquired pressure injuries (HAPIs) in acutely ill patients. Data from a randomized controlled trial, published literature, and existing cost-effectiveness publications were incorporated into the model, with sensitivity analyses performed.

Engelgeer et al^19^ used data from the “Promoting Implementation of Seizure Detection Devices in Epilepsy Care (PROMISE)” trial involving 60 children with nocturnal motor seizures. It evaluated the economic impacts from a societal perspective, considering health care and productivity costs. The comparison was made in a 2-month period without and using the NightWatch seizure detection device. The assessment was based on caregiver responses to questionnaires, gauging caregiver stress, quality of life, medical consumption, and productivity at the start and end of both periods.

The study by Brouwers et al^18^ was an economic evaluation of a randomized clinical trial involving 300 patients with coronary artery disease, comparing traditional center-based cardiac rehabilitation with cardiac telerehabilitation. The cardiac telerehabilitation group used a web-based application along with a heart rate monitor and a triaxial accelerometer for home-based training after 6 supervised sessions. The evaluation analyzed societal costs over 1 year.

Sangster et al^26^ analyzed 2 interventions based on social cognitive theory. The healthy weight group received brochures, a calendar for tracking goals, and a pedometer and participated in several phone-coaching sessions. Depending on their body mass index, they were advised on diet and varying physical activity levels. The physical activity group, aimed at cardiac patients, received a pedometer and step-recording calendar, and participated in physical activity coaching calls. Both groups received follow-up booster calls postintervention.

Roze et al^27^ used their “Center for Outcomes Research (CORE)” Diabetes Model to evaluate a sensor-augmented insulin pump vs self-monitoring of plasma glucose plus continuous subcutaneous insulin infusion in type 1 diabetes patients. It considered clinical benefits, cost-effectiveness, and health state utilities based on a published meta-analysis.

Jiang et al^24^ analyzed the cost-effectiveness of 3 strategies for patients needing implantable cardioverter-defibrillator (ICD) reimplantation: home without a wearable cardioverter-defibrillator (WCD), home with a WCD, or staying in the hospital. The outcomes measured over 8 weeks and a subsequent 5 years were mortality rates, medical costs, and QALYs. The model started with an 8-week decision tree followed by a 5-year Markov model to track outcomes yearly after ICD reimplantation.

Evers et al^23^ used a Markov model to study 3 strategies for pediatric dilated cardiomyopathy patients with a low ejection fraction preparing for ICD. Comparisons were made between patients staying in a telemetry unit or at home with or without WCD.

## Discussion

This systematic review revealed a diverse range of wearable devices used across various health care settings, demonstrating the broad applicability of these technologies in health management. The types of wearables encompassed in the included studies were respiratory rate monitors, pedometers, fall-prediction devices, patient monitoring systems for pressure injuries, seizure detection devices, cardiac monitoring and rehabilitation devices, and insulin therapy devices.

Differing methodologies in cost calculation across the studies, encompassing various types of expenses, could have influenced their outcomes.^28^ This variation also poses a challenge when attempting to conduct a meta-analysis. In addition, their level of cost-effectiveness varied significantly depending on the specific health care context and the standard of care against which they were compared. Some wearable devices, including pedometers and WCD, were found to be more cost-effective in specific populations or under certain conditions.^24^^,^^26^

### The Advent of Wearable Technologies in Health Care

Wearable technologies have ushered in a broad spectrum of tools designed to address various challenges in the health care sector. Further discussed is an overview of the wearable devices that have been explored in the studies encompassed by this systematic review.

#### Respiratory Rate Monitors

The monitoring of respiratory rate is critical for acutely ill and hospitalized patients, as it has been pointed out as the most accurate indicator of clinical deterioration and cardiac arrest risk.^29^, ^30^, ^31^, ^32^ Wearable technology for respiratory rate monitoring offers a significant advancement, enabling early detection of adverse health events while allowing patient mobility. This is particularly beneficial for individuals who are clinically stable yet at a high risk of deterioration. Wearable respiratory rate monitors use various technologies to accurately capture breathing-related movements and differentiate them from artifacts of walking and talking through accelerometers and detection algorithms.^33^ The integration of wearable respiratory rate monitors into clinical practice represents a shift toward proactive recovery, providing real-time data for early intervention, and potentially improving patient outcomes.^34^

#### Pedometers

Pedometers, simple wearable devices designed to count the number of steps taken by the wearer, have been extensively studied for their role in promoting physical activity and improving health outcomes. These devices operate by detecting motion, typically through a mechanical or electronic motion sensor, to estimate the distance walked or run. Despite its simplicity, the steps count as a parameter has been largely studied and reported its association with patient mortality.^35^ Research has also shown that interventions based on pedometer use can lead to significant health benefits, including increases in physical activity, reductions in body mass index, and decreased systolic blood pressure.^36^^,^^37^ With technological advancements, modern pedometers now offer features such as distance estimation, calorie tracking, and integration with smartphone applications, enhancing their utility and accuracy in monitoring physical activity.^38^ This evidence highlights the importance of pedometers in health promotion and the evolving capabilities of wearable technology to support active lifestyles.

#### Fall-Prediction Devices

Wearable fall-prediction systems leverage sensors and machine learning algorithms to assess the risk of falls in real-time. These devices, which can be worn on the wrist, waist, or as part of clothing, monitor users’ movements, gait patterns, and balance, providing alerts and data that can be used to prevent falls, especially in elderly populations and individuals with mobility impairments.

Recent studies have highlighted the effectiveness and technological advancements in wearable fall-prediction devices. For instance, a 2021 study by García-Villamil et al^39^ found the accuracy of a new wearable sensor system in detecting gait patterns that precede falls in older adults. Further research has explored the integration of artificial intelligence with wearable devices to predict fall risk with greater precision.^40^^,^^41^

#### Patient Monitoring Systems for Pressure Injuries

Pressure injuries pose a significant financial burden in the United States, with estimates exceeding $20 billion annually.^42^ A key strategy for preventing HAPIs is the implementation of patient repositioning protocols; however, adherence to these protocols remains a challenge.^43^ In response, monitoring technologies have been developed to mitigate the health care burden of HAPIs. A notable example included in this study is the LEAF system (Smith+Nephew), which monitors the orientation and activity of patients susceptible to pressure injuries. This system features a wearable sensor placed on the chest that monitors patient positioning and is connected to a device that both records and alerts health care staff about necessary repositioning movements. This system has been reported to increase adherence with turning protocols and to decrease the risk of HAPIs compared with traditional turn reminders and practices.^44^^,^^45^

#### Seizure Detection Devices

Wearable seizure detection devices represent a significant advancement in the real-time monitoring of individuals with epilepsy, providing crucial alerts to caregivers and medical professionals about seizure events. Their importance is further underscored by the fact that the absence of continuous supervision is linked to an increased risk of sudden unexpected death in epilepsy.^46^

Research has shown the sensitivity of these devices in detecting generalized tonic-clonic seizures, underscoring their potential to enhance epilepsy management.^47^^,^^48^ One notable example is the NightWatch system, which uses a combination of photoplethysmography and accelerometry. This system is designed to alert caregivers through alarms triggered by significant heart rate changes or continuous rhythmic movements, indicative of seizure activity.^48^

It is important to note that while these technologies are particularly recommended for the detection of generalized tonic-clonic seizures and focal-to-bilateral tonic-clonic seizures, there is a lack of evidence supporting their effectiveness in detecting other types of seizures.^49^

#### Cardiac Monitoring and Rehabilitation Devices

Wearable cardioverter-defibrillators are devices particularly valuable for patients who are at temporary high risk of sudden cardiac arrest but are not immediate candidates for an ICD due to various reasons, such as awaiting ICD implantation or undergoing medical treatment that might reverse their risk. Wearable cardioverter-defibrillators consist of a wearable garment with integrated sensing and defibrillation electrodes and a battery-powered monitor-defibrillator that records ECGs and detects arrhythmias, worn on a belt or strap.

Wearable cardioverter-defibrillators continuously monitor heart rhythm and can automatically deliver a defibrillation shock if a life-threatening heart rhythm is detected, thereby providing a critical bridge to more permanent interventions or during periods of transient risk.^50^ The clinical benefit of WCDs has been found, a meta-analysis reported that WCDs terminated ventricular tachycardia and ventricular fibrillation in >95% of patients requiring defibrillation.^51^

Cardiac telerehabilitation transforms traditional cardiac rehab by delivering it directly to patients’ homes through wearable devices and digital communication. This approach can boost participation and completion rates by overcoming common barriers, with wearables enabling continuous health monitoring and personalized care from a distance.^52^

#### Insulin Therapy Devices

Wearable insulin therapy devices, such as continuous glucose monitors and insulin pumps, have emerged as promising tools to revolutionize the management of diabetes. Continuous glucose monitors provide continuous tracking of glucose levels through a sensor placed under the skin, offering real-time data and alerts for significant fluctuations. Insulin pumps administer insulin through a subcutaneous catheter, allowing for tailored dosing based on current needs, often using data from continuous glucose monitors. Together, these devices create an artificial pancreas system, automating insulin delivery based on glucose levels.^53^ Research highlights their effectiveness in improving glycemic control and reducing risks associated with glucose level fluctuations.^54^^,^^55^ Furthermore, wearable artificial pancreas systems have shown superiority when compared with the use of insulin pumps alone.^56^^,^^57^

### Summary of Findings

#### Key Findings Overview

The data analyzed in multiple studies show that wearable technologies can be cost-effective across various health care scenarios.^18^, ^19^, ^20^, ^21^, ^22^, ^23^, ^24^, ^25^, ^26^, ^27^

The effectiveness of wearables, often measured in QALYs, generally appears favorable, with many interventions leading to improvements in QALYs compared with standard care. This pattern has been previously shown with other digital health interventions, such as telehealth interventions and virtual reality cognitive behavioral therapy.^58^^,^^59^

It is worth mentioning that certain studies observed lower QALYs among wearables user groups. However, despite this finding, the ICER remained higher owing to reduced health care expenses.^18^^,^^19^^,^^23^

The cost of wearable health care technologies varied significantly depending on the context. Some studies found that wearable devices led to overall cost savings,^19^^,^^21^^,^^22^ whereas others indicated cost increments but were still deemed cost-effective owing to their high impact on health outcomes.^20^^,^^23^^,^^25^^,^^27^

It was also observed in a case that cost-effectiveness was not easily achieved. Jiang et al^24^ reported that, compared with hospital stay, the use of WCD at home in patients requiring ICD reimplantation was deemed cost-saving at a daily cost of $72. However, at a higher daily cost of $96, WCD usage lost its cost-effectiveness.^24^ This underscores the need for careful economic evaluation before introducing wearables into routine care, factoring in local costs and willingness-to-pay thresholds.

#### Trends and Patterns

Notably, some wearable interventions such as RespiraSense^22^ and LEAF Patient Monitoring System^21^ were found to be overall dominant strategies, being both more effective and less costly than the alternatives. Regarding cost-effectiveness thresholds or willingness-to-pay, there was a notable variance across different countries, which impacted the evaluation of interventions. The differences in these thresholds can significantly influence the perceived value of a health care intervention and its likelihood of being adopted.^60^^,^^61^ Certain countries explicitly state cost-effectiveness thresholds, such as United Kingdom’s National Institute for Health and Care Excellence, which sets a range of £20,000–30,000 for appraising technologies. Conversely, this threshold is implicitly determined in other countries through negotiations between technology providers and payers.^62^

Another pattern observed is that wearable technology appears to have a moderate to high probability of being cost-effective in many scenarios, ranging from 66% to 100% in the studies examined, suggesting a strong potential for implementing such technology in health care. Others did not report this parameter,^20^^,^^23^ underscoring a gap in current reporting practices that future studies could aim to address.

Although our systematic review did encompass studies with varying time horizons, economic perspectives, and cost-effectiveness ratios, directly comparing these studies was challenging owing to the diversity of the interventions analyzed. Moreover, it is worth citing Kim et al,^63^ who reported that the choice of time horizon in cost-effectiveness analyses significantly influenced the perceived value of medical interventions. Using a 5-year cutoff for short and long-term horizons, they observed that longer time horizons generally yielded more favorable ICERs. This highlights the importance of choosing adequate time horizons to capture all potential consequences of interventions.^63^

### Health Care Policy and Resource Allocation

The studies in this review presented compelling evidence of cost-effectiveness in wearable technology, indicating the potential for substantial savings or outcomes improvement in health care expenditure, supporting investments in such technology to optimize resource allocation.

Furthermore, Roze et al^27^ reported that sensor-augmented pump insulin therapy could reduce diabetes-related complications and increase life expectancy. This implies the necessity for policies supporting technologies that enhance patient outcomes, potentially reducing the burden on health services in the long-term.

On the contrary, although wearable devices offer health care advancements, they can also inadvertently increase anxiety and unnecessary medical interventions in patients susceptible to excessive symptom monitoring, such as those with intermittent atrial fibrillation.^64^ This can negatively impact their psychological well-being and lead to overusing health care resources.

Considering the ubiquity of wearable technologies in health care, the policy should underscore the importance of robust data privacy standards. These regulations must guarantee the safe use and protection of personal health data, preventing unauthorized access or misuse while remaining adaptable to the continuously evolving wearable technology landscape.^65^, ^66^, ^67^

### Clinical Practice Adaptations

Continuous monitoring in hospital inpatient care has been standard for years using traditional patient vital signs monitors.^68^ However, monitoring nonbedridden patients presents challenges owing to the bulky nature of traditional devices.^69^^,^^70^ The advent of wireless wearable sensors, capable of tracking vital signs and additional parameters such as movement and sleep quality, offers enhanced patient monitoring in clinical settings, improving patient comfort and mobility.^71^^,^^72^

The evidence presented suggests several potential adaptations to clinical practices. Javanbakht et al^22^ highlighted the utility of automatic respiratory rate monitoring in pneumonia patients over intermittent nurse-led monitoring, suggesting a re-evaluation of monitoring strategies in hospitals.^22^

The integration of smartwatches into health care holds promise for early detection, prevention, and improved management of diverse medical conditions.^73^ For example, smartwatches currently have the capability to track sleep patterns. These data could potentially be used to predict the onset or exacerbation of schizophrenia symptoms, thereby aiding in the prevention of relapses.^74^ Moreover, smartwatches can provide continuous objective measures for conditions like Parkinson disease, including arm swing, tremor duration, and finger tapping.^75^

Current smartwatches with single-lead ECG functionality can effectively identify cardiac dysfunction. In a study by Attia et al,^76^ an artificial intelligence algorithm analyzed smartwatch ECGs with a high accuracy level, achieving an area under the curve of 0.885 for detecting low ejection fraction.^76^

**Figure 4:**
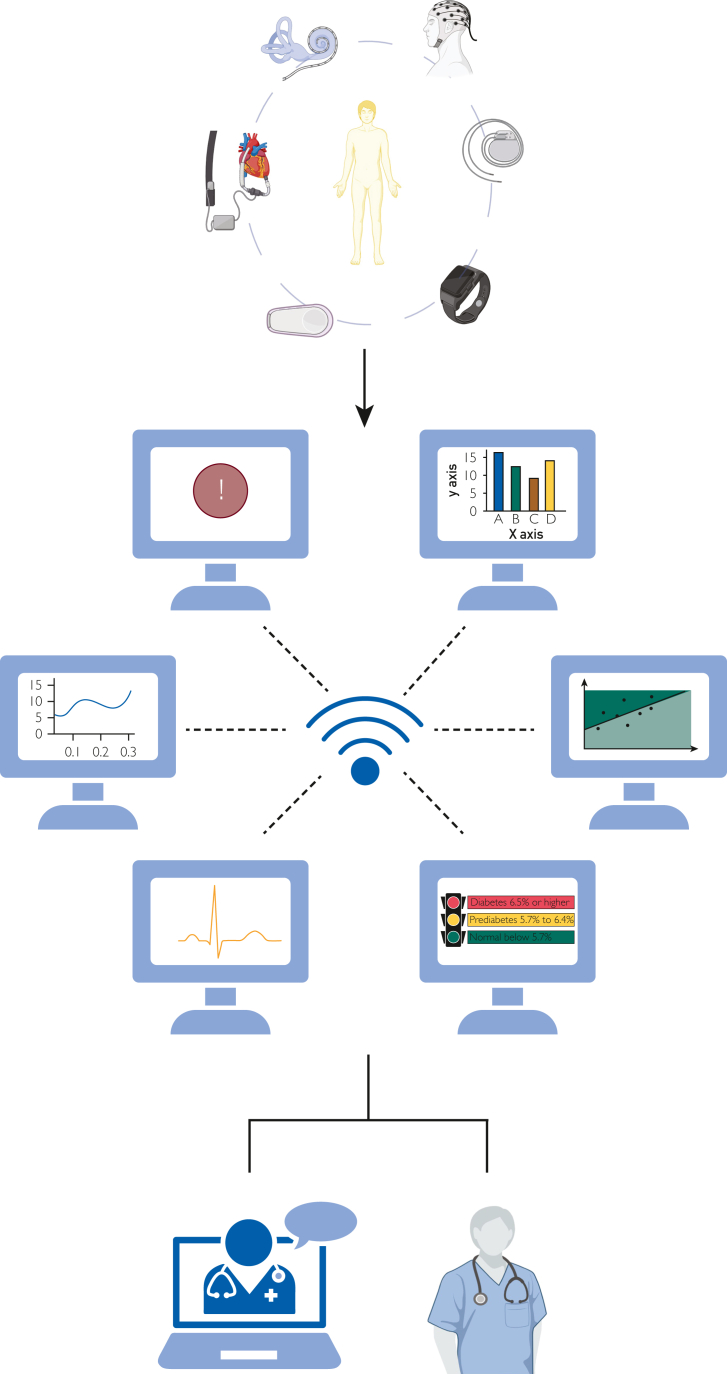
Integration of wearable health devices in health care systems: the utilization of various wearable health devices, from heart monitors to insulin sensors, and their interconnection with digital platforms for data analysis. Data gathered from these devices are transmitted to central systems, allowing real-time monitoring and immediate medical response. This integration offers potential cost savings, improved patient monitoring, and better health outcomes.

These applications represent just a few examples of the potential of wearables in health care, although the full range of possibilities is broad and continually expanding.^67^^,^^77^ For successful integration of wearable advancements, meticulous planning and professional training are required (Figure 4). Health care providers must be proficient in using and interpreting data from these devices while effectively using their diverse applications.^78^

### Limitations

#### Limitations of Included Studies

The studies presented highlight several common limitations. The lack of robust clinical evidence informing their analyses was a common concern, as highlighted by some authors.^20^, ^21^, ^22^ This highlights a need for more comprehensive clinical trials within their respective fields.

Another recurrent theme across studies is methodological constraints. Authors like Over et al^25^ and Nherera et al^21^ point out that their models made use of simplifying assumptions, which might not adequately capture real-world complexities. Jiang et al^24^ also echo this sentiment, mentioning their study’s simplified model design.

Cost-effectiveness analyses by some authors were conducted over a relatively short duration.^18^^,^^19^^,^^23^ These condensed timeframes might not capture the full extent of long-term effects, potentially skewing the results. Moreover, several authors recognized that their models do not include indirect costs or broader societal impacts, which could undervalue the estimated costs or cost-effectiveness of the interventions under study.^22^^,^^24^^,^^25^

The limited generalizability of results is also a concern across studies owing to specific study settings and population demographic characteristics. This concern is explicitly stated in a few studies. In addition, potential bias from self-reported questionnaires in the studies of Sangster et al^26^ and Brouwers et al^18^ could distort the actual consumption of health care resources.^67^

Finally, some studies, like Sangster et al,^26^ lacked a nonwearable group comparator, making it challenging to interpret the relative value of the interventions.^26^ Furthermore, Jiang et al^24^ raised the issue of language bias, with non-English literature being ignored, with the consequent lack of generalizable results for China.^24^

#### Conflicts of Interests

Conflicts of Interest varied across the studies in our systematic review. Four studies^19^^,^^21^^,^^22^^,^^27^ reported an explicit conflict of interest; moreover, their findings were favorable toward the specific wearable technologies being studied. Conversely, 5 studies reported no conflicts of interest.^18^^,^^20^^,^^24^, ^25^, ^26^ The significant presence of conflicts of interest among the included studies on the cost-effectiveness of wearable devices emphasizes the need for careful interpretation, as such conflicts can introduce biases and undermine the credibility of research findings.^79^

On the contrary, Evers et al^23^ did not provide any information on conflicts of interest, introducing a degree of uncertainty. Future research should maintain transparency in declaring conflicts of interest to foster credibility and reliability in results.

#### Limitations of This Review

This systematic review has a few potential limitations. First, our search scope was limited to certain databases and specific keywords, potentially omitting relevant studies. Second, we included only English-language publications, disregarding potentially pertinent research in other languages. Finally, records with vague details, such as conference abstracts, were disregarded, potentially skewing the results.

### Implications for Future Research

In light of the findings from our systematic review, several areas warrant further investigation. Primarily, our review underscores a need for expanded research on the cost-effectiveness of wearable devices in health care. Although our search strategy yielded studies on various health conditions and wearable types, more research is required to broaden the knowledge base, particularly for less-studied conditions and emerging wearable technologies.

Moreover, the cost-effectiveness of wearables varies across different health care settings.^26^ Hence, future studies could explore this aspect more comprehensively. The insights generated would assist in tailoring the deployment of wearable devices, ensuring they are used optimally within different health care contexts.^80^

In addition, we observed significant variations in the cost-effectiveness thresholds across different countries, underscoring the need for more research on context-specific cost-effectiveness thresholds. Such thresholds, although accommodating the socioeconomic conditions of each country, would make it easier to compare cost-effectiveness studies across different jurisdictions and foster international collaboration.^81^^,^^82^

It is also essential to underscore the potential value of research into the long-term cost-effectiveness of wearable devices in health care. In cost-effectiveness studies, careful selection of the time horizon for assessing outcomes is crucial. It should be sufficient to account for the intended and unintended effects of the interventions; however, in some cases, longer time horizons can add unnecessary cost and complexity.^83^

## Conclusion

The cost-effectiveness of wearable technologies is context-specific and dependent on several factors, including the type of wearable, the health condition being managed, local cost structures, willingness-to-pay thresholds, and several others. These aspects must be thoroughly evaluated to make informed decisions about incorporating wearable technology in health care.

The future research agenda should aim further to illuminate the cost-effectiveness landscape of wearable devices in health care in order to strengthen the evidence base needed by health care providers, policymakers, and patients to make informed decisions. Finally, although wearable technology shows promise in delivering cost-effective health care, more research is necessary to understand the long-term benefits and potential risks associated with these devices.

## Potential Competing Interests

Given their role as Editorial Board Members, Dr Bart Demaerschalk and Dr Michael Maniaci, had no involvement in the peer-review of this article and have no access to information regarding its peer-review.

## Declaration of AI and AI-assisted technologies in the writing process

During the preparation of this work, the authors used OpenAI-ChatGPT in order to improve English clarity, proficiency, and grammar enhancement. After using this tool, the authors reviewed and edited the content as needed and takes full responsibility for the content of the publication.

## Supplemental Online Material

Appendix 1

PRISMA 2020 checklist

Statement

## References

[1] Shin G., Jarrahi M.H., Fei Y. (2019). Wearable activity trackers, accuracy, adoption, acceptance and health impact: a systematic literature review. J Biomed Inform.

[2] Piwek L., Ellis D.A., Andrews S., Joinson A. (2016). The rise of consumer health wearables: promises and barriers. PLOS Med.

[3] Finni T., Hu M., Kettunen P., Vilavuo T., Cheng S. (2007). Measurement of EMG activity with textile electrodes embedded into clothing. Physiol Meas.

[4] Kim J., Campbell A.S., de Ávila B.E.-F., Wang J. (2019). Wearable biosensors for healthcare monitoring. Nat Biotechnol.

[5] Strik M., Ploux S., Weigel D. (2024). The use of smartwatch electrocardiogram beyond arrhythmia detection. Trends Cardiovasc Med.

[6] Ferguson T., Olds T., Curtis R. (2022). Effectiveness of wearable activity trackers to increase physical activity and improve health: a systematic review of systematic reviews and meta-analyses. Lancet Digit Health.

[7] Majumder S., Mondal T., Deen M.J. (2017). Wearable sensors for remote health monitoring. Sensors (Basel).

[8] Sanders G.D., Neumann P.J., Basu A. (2016). Recommendations for conduct, methodological practices, and reporting of cost-effectiveness analyses: second panel on cost-effectiveness in health and medicine. JAMA.

[9] Sanders G.D., Maciejewski M.L., Basu A. (2019). Overview of cost-effectiveness analysis. JAMA.

[10] Boriani G., Mantovani L.G., Cortesi P.A. (2021). Cost-minimization analysis of a wearable cardioverter defibrillator in adult patients undergoing ICD explant procedures: clinical and economic implications. Clin Cardiol.

[11] Mattison G., Canfell O., Forrester D. (2022). The influence of wearables on health care outcomes in chronic disease: systematic review. J Med Internet Res.

[12] Colquitt J.L., Jones J., Harris P. (2011). Bone-anchored hearing aids (BAHAs) for people who are bilaterally deaf: a systematic review and economic evaluation. Health Technol Assess.

[13] Bruchhage K.L., Leichtle A., Schönweiler R. (2017). Systematic review to evaluate the safety, efficacy and economical outcomes of the Vibrant Soundbridge for the treatment of sensorineural hearing loss. Eur Arch Otorhinolaryngol.

[14] Chao T.-K., Chen T.H.-H. (2008). Cost-effectiveness of hearing aids in the hearing-impaired elderly: a probabilistic approach. Otol Neurotol.

[15] Taylor R.S., Paisley S., Davis A. (2001). Systematic review of the clinical and cost effectiveness of digital hearing aids. Br J Audiol.

[16] Page M.J., Moher D., Bossuyt P.M. (2021). PRISMA 2020 explanation and elaboration: updated guidance and exemplars for reporting systematic reviews. BMJ.

[17] Sterne J.A., Hernán M.A., Reeves B.C. (2016). ROBINS-I: a tool for assessing risk of bias in non-randomised studies of interventions. BMJ.

[18] Brouwers R.W.M., van der Poort E.K.J., Kemps H.M.C., van den Akker-van Marle M.E., Kraal J.J. (2021). Cost-effectiveness of cardiac telerehabilitation with relapse prevention for the treatment of patients with coronary artery disease in the Netherlands. JAMA Netw Open.

[19] Engelgeer A., van Westrhenen A., Thijs R.D., Evers S.M.A.A. (2022). An economic evaluation of the NightWatch for children with refractory epilepsy: insight into the cost-effectiveness and cost-utility. Seizure.

[20] Federici C., Pecchia L. (2021). Early health technology assessment using the MAFEIP tool. A case study on a wearable device for fall prediction in elderly patients. Health Technol.

[21] Nherera L., Larson B., Cooley A., Reinhard P. (2021). An economic analysis of a wearable patient sensor for preventing hospital-acquired pressure injuries among the acutely ill patients. Int J Health Econ Manag.

[22] Javanbakht M., Moradi-Lakeh M., Mashayekhi A., Atkinson J. (2022). Continuous monitoring of respiratory rate with wearable sensor in patients admitted to hospital with pneumonia compared with intermittent nurse-led monitoring in the United Kingdom: a cost-utility analysis. Pharmacoecon Open.

[23] Evers P.D., Anderson J.B., Ryan T.D., Czosek R.J., Knilans T.K., Spar D.S. (2020). Wearable cardioverter-defibrillators in pediatric cardiomyopathy: a cost-utility analysis. Heart Rhythm.

[24] Jiang X., Ming W.K., You J.H.S. (2019). Potential cost-effectiveness of wearable cardioverter-defibrillator for patients with implantable cardioverter-defibrillator explant in a high-income city of China. J Cardiovasc Electrophysiol.

[25] Over E.A., Wendel-Vos G.W., van den Berg M. (2012). Cost-effectiveness of counseling and pedometer use to increase physical activity in the Netherlands: a modeling study. Cost Eff Resour Alloc.

[26] Sangster J., Church J., Haas M., Furber S., Bauman A. (2015). A comparison of the cost-effectiveness of two pedometer-based telephone coaching programs for people with cardiac disease. Heart Lung Circ.

[27] Roze S., Saunders R., Brandt A.S., de Portu S., Papo N.L., Jendle J. (2015). Health-economic analysis of real-time continuous glucose monitoring in people with type 1 diabetes. Diabet Med.

[28] Ryder H.F., McDonough C., Tosteson A.N.A., Lurie J.D. (2009). Decision analysis and cost-effectiveness analysis. Semin Spine Surg.

[29] Churpek M.M., Yuen T.C., Huber M.T., Park S.Y., Hall J.B., Edelson D.P. (2012). Predicting cardiac arrest on the wards: a nested case-control study. Chest.

[30] De Sario Velasquez G.D., Forte A.J., McLeod C.J. (2023). Predicting cardiopulmonary arrest with digital biomarkers: a systematic review. J Clin Med.

[31] Fieselmann J.F., Hendryx M.S., Helms C.M., Wakefield D.S. (1993). Respiratory rate predicts cardiopulmonary arrest for internal medicine inpatients. J Gen Intern Med.

[32] Lam T., Mak P., Siu W., Lam M., Cheung T., Rainer T. (2006). Validation of a modified early warning score (MEWS) in emergency department observation ward patients. HK J Emerg Med.

[33] Subbe C.P., Kinsella S. (2018). Continuous monitoring of respiratory rate in emergency admissions: evaluation of the RespiraSense™ sensor in acute care compared to the industry standard and gold standard. Sensors (Basel).

[34] Leenen J.P.L., Leerentveld C., van Dijk J.D., van Westreenen H.L., Schoonhoven L., Patijn G.A. (2020). Current evidence for continuous vital signs monitoring by wearable wireless devices in hospitalized adults: systematic review. J Med Internet Res.

[35] Stens N.A., Bakker E.A., Mañas A. (2023). Relationship of daily step counts to all-cause mortality and cardiovascular events. J Am Coll Cardiol.

[36] Bravata D.M., Smith-Spangler C., Sundaram V. (2007). Using pedometers to increase physical activity and improve health: a systematic review. JAMA.

[37] Tudor-Locke C., Bassett D.R. (2004). How many steps/day are enough? Preliminary pedometer indices for public health. Sports Med.

[38] Lee J.M., Kim Y., Welk G.J. (2014). Validity of consumer-based physical activity monitors. Med Sci Sports Exerc.

[39] García-Villamil G., Neira-Álvarez M., Huertas-Hoyas E., Ramón-Jiménez A., Rodríguez-Sánchez C. (2021). A pilot study to validate a wearable inertial sensor for gait assessment in older adults with falls. Sensors (Basel).

[40] Kiprijanovska I., Gjoreski H., Gams M. (2020). Detection of gait abnormalities for fall risk assessment using wrist-worn inertial sensors and deep learning. Sensors (Basel).

[41] Rehman R.Z.U., Zhou Y., Del Din S. (2020). Gait analysis with wearables can accurately classify fallers from non-fallers: a step toward better management of neurological disorders. Sensors (Basel).

[42] Padula W.V., Delarmente B.A. (2019). The national cost of hospital-acquired pressure injuries in the United States. Int Wound J.

[43] Reddy M., Gill S.S., Rochon P.A. (2006). Preventing pressure ulcers: a systematic review. JAMA.

[44] Pickham D., Berte N., Pihulic M., Valdez A., Mayer B., Desai M. (2018). Effect of a wearable patient sensor on care delivery for preventing pressure injuries in acutely ill adults: a pragmatic randomized clinical trial (LS-HAPI study). Int J Nurs Stud.

[45] Yap T.L., Kennerly S.M., Ly K. (2019). Pressure injury prevention: outcomes and challenges to use of resident monitoring technology in a nursing home. J Wound Ostomy Continence Nurs.

[46] Sveinsson O., Andersson T., Mattsson P., Carlsson S., Tomson T. (2020). Clinical risk factors in SUDEP: a nationwide population-based case-control study. Neurology.

[47] Beniczky S., Conradsen I., Henning O., Fabricius M., Wolf P. (2018). Automated real-time detection of tonic-clonic seizures using a wearable EMG device. Neurology.

[48] van Westrhenen A., Lazeron R.H.C., van Dijk J.P., Leijten F.S.S., Thijs R.D., Dutch TeleEpilepsy Consortium (2023). Multimodal nocturnal seizure detection in children with epilepsy: a prospective, multicenter, long-term, in-home trial. Epilepsia.

[49] Beniczky S., Wiebe S., Jeppesen J. (2021). Automated seizure detection using wearable devices: a clinical practice guideline of the International League Against Epilepsy and the International Federation of Clinical Neurophysiology. Epilepsia.

[50] Piccini J.P., Allen L.A., Kudenchuk P.J. (2016). Wearable cardioverter-defibrillator therapy for the prevention of sudden cardiac death: a science advisory from the American Heart Association. Circulation.

[51] Nguyen E., Weeda E.R., Kohn C.G. (2018). Wearable cardioverter-defibrillators for the prevention of sudden cardiac death: a meta-analysis. J Innov Card Rhythm Manag.

[52] Brouwers R.W.M., Scherrenberg M., Kemps H.M.C., Dendale P., Snoek J.A. (2024). Cardiac telerehabilitation: current status and future perspectives. Neth Heart J.

[53] Pinnaro C.T., Tansey M.J. (2021). The evolution of insulin administration in type 1 diabetes. J Diabetes Mellitus.

[54] Haidar A., Legault L., Messier V., Mitre T.M., Leroux C., Rabasa-Lhoret R. (2015). Comparison of dual-hormone artificial pancreas, single-hormone artificial pancreas, and conventional insulin pump therapy for glycaemic control in patients with type 1 diabetes: an open-label randomised controlled crossover trial. Lancet Diabetes Endocrinol.

[55] Russell S.J., Beck R.W., Bionic Pancreas Research Group (2022). Multicenter, randomized trial of a bionic pancreas in type 1 diabetes. N Engl J Med.

[56] Russell S.J., El-Khatib F.H., Sinha M. (2014). Outpatient glycemic control with a bionic pancreas in type 1 diabetes. N Engl J Med.

[57] Russell S.J., Hillard M.A., Balliro C. (2016). Day and night glycaemic control with a bionic pancreas versus conventional insulin pump therapy in preadolescent children with type 1 diabetes: a randomised crossover trial. Lancet Diabetes Endocrinol.

[58] Bergmo T.S. (2014). Using QALYs in telehealth evaluations: a systematic review of methodology and transparency. BMC Health Serv Res.

[59] Pot-Kolder R., Veling W., Geraets C. (2020). Cost-effectiveness of virtual reality cognitive behavioral therapy for psychosis: health-economic evaluation within a randomized controlled trial. J Med Internet Res.

[60] Brouwer W., van Baal P., van Exel J., Versteegh M. (2019). When is it too expensive? Cost-effectiveness thresholds and health care decision-making. Eur J Health Econ.

[61] McDougall J.A., Furnback W.E., Wang B.C.M., Mahlich J. (2020). Understanding the global measurement of willingness to pay in health. J Mark Access Health Policy.

[62] Thokala P., Ochalek J., Leech A.A., Tong T. (2018). Cost-effectiveness thresholds: the past, the present and the future. Pharmacoeconomics.

[63] Kim D.D., Wilkinson C.L., Pope E.F., Chamber J.D., Cohen J.T., Neumann P.J. (2017). The influence of time horizon on results of cost-effectiveness analyses. Expert Rev Pharmacoecon Outcomes Res.

[64] Rosman L., Gehi A., Lampert R. (2020). When smartwatches contribute to health anxiety in patients with atrial fibrillation. CardioVasc Digit Health J.

[65] Brinson N.H., Rutherford D.N. (2020). Privacy and the quantified self: a review of U.S. health information policy limitations related to wearable technologies. J Con Aff.

[66] Ajana B. (2017). Digital health and the biopolitics of the Quantified Self. Digit Health.

[67] Dunn J., Runge R., Snyder M. (2018). Wearables and the medical revolution. Pers Med.

[68] Guidelines for intensive care unit admission, discharge, and triage (1999). Task Force of the American College of Critical Care Medicine, Society of Critical Care Medicine. Crit Care Med.

[69] Banks J., McArthur J., Gordon G. (2000). Flexible monitoring in the management of patient care processes: a pilot study. Lippincotts Case Manag.

[70] Bonnici T., Tarassenko L., Clifton D.A., Watkinson P. (2013). The digital patient. Clin Med (Lond).

[71] Casson AJ, Smith S, Duncan JS, Rodriguez-Villegas E, eds. Wearable EEG: what is it, why is it needed and what does it entail? *Annu Int Conf IEEE Eng Med Biol Soc*. 30th Annual International Conference of the IEEE Engineering in Medicine and Biology Society. 2008; 2008;(August 20 to 25):5867-5870. 10.1109/IEMBS.2008.4650549 19164052

[72] Kroll R.R., McKenzie E.D., Boyd J.G. (2017). Use of wearable devices for post-discharge monitoring of ICU patients: a feasibility study. J Intensive Care.

[73] Lu T.-C., Fu C.-M., Ma M.H.-M., Fang C.-C., Turner A.M. (2016). Healthcare applications of smart watches. A systematic review. Appl Clin Inform.

[74] Fonseka L.N., Woo B.K.P. (2022). Wearables in schizophrenia: update on current and future clinical applications. JMIR MHealth UHealth.

[75] Adams J.L., Kangarloo T., Tracey B. (2023). Using a smartwatch and smartphone to assess early Parkinson’s disease in the WATCH-PD study. npj Parkinsons Dis.

[76] Attia Z.I., Harmon D.M., Dugan J. (2022). Prospective evaluation of smartwatch-enabled detection of left ventricular dysfunction. Nat Med.

[77] Lu L., Zhang J., Xie Y. (2020). Wearable health devices in health care: narrative systematic review. JMIR MHealth UHealth.

[78] Dias D., Paulo Silva Cunha J. (2018). Wearable health devices-vital sign monitoring, systems and technologies. Sensors (Basel).

[79] Ioannidis J.P.A. (2005). Why most published research findings are false. PLOS Med.

[80] Smuck M., Odonkor C.A., Wilt J.K., Schmidt N., Swiernik M.A. (2021). The emerging clinical role of wearables: factors for successful implementation in healthcare. npj Digit Med.

[81] Bertram M.Y., Lauer J.A., De Joncheere K. (2016). Cost-effectiveness thresholds: pros and cons. Bull World Health Organ.

[82] Woods B., Revill P., Sculpher M., Claxton K. (2016). Country-level cost-effectiveness thresholds: initial estimates and the need for further research. Value Health.

[83] Basu A., Maciejewski M.L. (2019). Choosing a time horizon in cost and cost-effectiveness analyses. JAMA.

