# Supplementary material for: Economic Perspective of the Use of Wearables in Health Care: A Systematic Review
Source: Mayo Clin Proc Digit Health. 2024 May 14;2(3):299–317. doi: 10.1016/j.mcpdig.2024.05.003 (PMC11975836; doi:10.1016/j.mcpdig.2024.05.003)
Supplement: Appendix 1 [file mmc1.pdf]

Appendix 1 - Search Strategy

PubMed: search performed on 03/28/2023 yielding 194 results.

("Costs and Cost Analysis"[Mesh]) AND "Wearable Electronic Devices"[Mesh]

EMBASE/MEDLINE: search performed on 03/28/2023 yielding 171 results.

1	exp wearable computer/ or exp wearable sensor/	10186
2	exp "cost effectiveness analysis"/	178837
3	1 and 2	171

CINAHL: search performed on 03/28/2023 yielding 139 results.

((MH "Costs and Cost Analysis+")) AND (("wearable") OR ((MH "Wearable Sensors+")))

Google Scholar: search performed on 03/28/2023 yielding 194 results. Only the 10 first pages were screened (first 100 articles).

"Cost effectiveness" AND "Wearable Electronic Devices"
