# Supplementary material for: Economic Perspective of the Use of Wearables in Health Care: A Systematic Review
Source: Mayo Clin Proc Digit Health. 2024 May 14;2(3):299–317. doi: 10.1016/j.mcpdig.2024.05.003 (PMC11975836; doi:10.1016/j.mcpdig.2024.05.003)
Supplement: Statement [file mmc3.docx]

Gioacchino D. De Sario Velasquez: Conceptualization, Methodology, Writing - Original Draft.

Sahar Borna: Validation, Investigation, Writing - Original Draft.

Michael J. Maniaci: Methodology, Data Curation, Writing - Review & Editing.

Jordan D. Coffey: Writing - Review & Editing, Project administration, Investigation.

Clifton R Haider: Data Curation, Validation, Writing - Review & Editing.

Bart M. Demaerschalk: Writing - Original Draft, Methodology, Writing - Review & Editing.

Antonio J. Forte: Conceptualization, Writing - Review & Editing, Supervision.
